# Cholinergic relevant functional reactivity is associated with dopamine responsiveness of tremor in Parkinson’s disease

**DOI:** 10.1007/s11682-021-00610-9

**Published:** 2022-01-01

**Authors:** Jingjing Wu, Cheng Zhou, Tao Guo, Xiaojun Guan, Ting Gao, Xueqin Bai, Haoting Wu, Jingwen Chen, Jiaqi Wen, Xiaocao Liu, Luyan Gu, Zhe Song, Min Xuan, Quanquan Gu, Peiyu Huang, Jiali Pu, Baorong Zhang, Xiaojun Xu, Minming Zhang

**Affiliations:** 1 Department of Radiology, The Second Affiliated Hospital, Zhejiang University School of Medicine, No.88 Jiefang Road, Shangcheng District, Hangzhou, 310009 China; 2 Department of Neurology, The Second Affiliated Hospital, Zhejiang University School of Medicine, Hangzhou, 310009 China

**Keywords:** Parkinson’s disease, Tremor, Dopamine responsiveness, fMRI, Basal forebrain

## Abstract

Tremor in Parkinson’s disease (PD) has distinct responsiveness to dopamine, which is supposed not be exclusively related to dopamine deficiency but has a close relationship with cholinergic system. This phenomenon indicates that cholinergic system may be an important regulatory for distinct dopamine responsiveness of parkinsonian tremor. Through investigating the alterations of cholinergic and dopaminergic network during levodopa administration, we aimed at exploring the mechanisms of differed dopamine responsiveness of parkinsonian tremor. Fifty-two PD patients with tremor were enrolled. MRI scanning, UPDRS III and its sub-symptom scores were collected in OFF and ON status (dopaminergic challenge test). Then, patients were divided into two groups (dopamine-resistant tremor and dopamine-responsive tremor) according to the tremor change rate median score. Dopaminergic and cholinergic network were obtained. LASSO regression was conducted to identify functional connectivity with distinct reactivity during levodopa administration between groups. Afterwards, detailed group comparisons, interaction and correlation analyses were performed. The reactivity of cholinergic connectivity showed the highest possibility to distinguish two groups, especially connectivity of right basal forebrain 123 to right parietal operculum cortex (R.BF123-R.PO). After levodopa administration, connectivity of R.BF123-R.PO was decreased for dopamine-responsive tremor while which remained unchanged for dopamine-resistant tremor. The reactivity of R.BF123-R.PO was negatively correlated with tremor change rate. Reduced cholinergic connectivity to parietal operculum may be an underlying mechanism for the responsive tremor in PD and the distinct cholinergic reactivity of parietal operculum to levodopa may be a core pathophysiology for the differed DA responsiveness of tremor in PD.

## Introduction

Parkinson’s disease (PD) is pathologically characterized by the nigrostriatal dopamine (DA) depletion, leading to the classic motor symptoms, including tremor, bradykinesia, and rigidity (Kalia & Lang, 2015; Kish et al., 1988). While the dopaminergic medication is efficient for bradykinesia and rigidity, the effect of which on tremor varies greatly among patients (Koller, 1986; Koller & Hubble, 1990; Zach et al., 2020), arguing that parkinsonian tremor has different phenotypes exhibiting as DA-responsive tremor and DA-resistant tremor (Zach et al., 2020). Currently, the mechanism underlying such heterogeneous treatment responsiveness of tremor remains unknown, limiting the development of adaptive treatment for DA-resistant tremor.

PD is a multi-neurotransmitter involved disease (Konig et al., 2019; Lim et al., 2009). The core parkinsonian symptoms are related to both dopaminergic and cholinergic mechanisms, in which tremor is especially suggested to be closely associated with the cholinergic system (Cantello et al., 1986; Koller, 1986; Pirker, 2003). It is reported that when tremor is the most prominent symptom for PD patients, anticholinergics may be particularly applicated (Connolly & Lang, 2014). Specifically, although both the acetylcholine (ACh) and DA are decreased in PD, DA deficiency accompanies a smaller ACh reduction, which results in ACh overactive relative to DA. Thus, an imbalance between these two kinds of neurotransmitters occurs, which contributes to the motor dysfunctions in PD (McKinley et al., 2019). The interactions between DA and ACh are rather complicated. The release of ACh from cholinergic neurons is regulated through both dopaminergic and cholinergic receptors (Konig et al., 2019), and the cholinergic neuronal activity is regulated by the DA application (Napier & Potter, 1989). Therefore, when levodopa, a DA precursor protein and the most widely used symptomatic PD drug (Cotzias et al., 1967; Hauser, 2009; Nutt, 2008), is administrated to PD patients, a different functional reactivity of dopaminergic and cholinergic systems would occur (Konig et al., 2019; Napier & Potter, 1989), which may be one of the mechanisms of the differed DA responsiveness of tremor among PD patients.

Resting-state functional Magnetic Resonance Imaging (rs-fMRI) detects the intrinsic or spontaneous brain fluctuations of the blood oxygen level-dependent signal (Cohen et al., 2008; Fox & Raichle, 2007), which provides a powerful approach to investigate the functional variations and explore the neurotransmitter-related alterations. The functional connectivity (FC) between basal ganglia and cerebral cortex is an indirect index of dopaminergic activity and is verified to be related to the clinical severity of PD (Dong et al., 2021; Montgomery, 2015; Obeso et al., 2000; Rolinski et al., 2015, 2016). Basal forebrain (BF) provides the principal source of ACh for cerebral cortex in brain (Geula & Slevin, 1989; Mesulam, 2013; Sparks et al., 1986). The FC between BF and cerebral cortex could be used to reflect cholinergic function (Li et al., 2014; Li et al., 2017). Previous studies suggested that the cholinergic network of BF was disrupted in PD (Kim et al., 2017; Lee et al., 2018). Taken together, by employing rs-fMRI, we could explore the distinct functional patterns of dopaminergic and cholinergic connectivity in PD patients with different types of tremor responses when levodopa is administrated, which could help reveal the mechanism of differed DA responsiveness of tremor and promote future therapeutic strategies for PD.

To sum up, this study aimed at investigating the alterations of cholinergic and dopaminergic connectivity during levodopa administration in PD. We hypothesized that the distinct dopaminergic or cholinergic functional reactivity to levodopa may be an underlying mechanism for the differed DA responsiveness of tremor in PD.

## Materials and methods

### Subjects and clinical variables acquisition

**Fig. 1:**
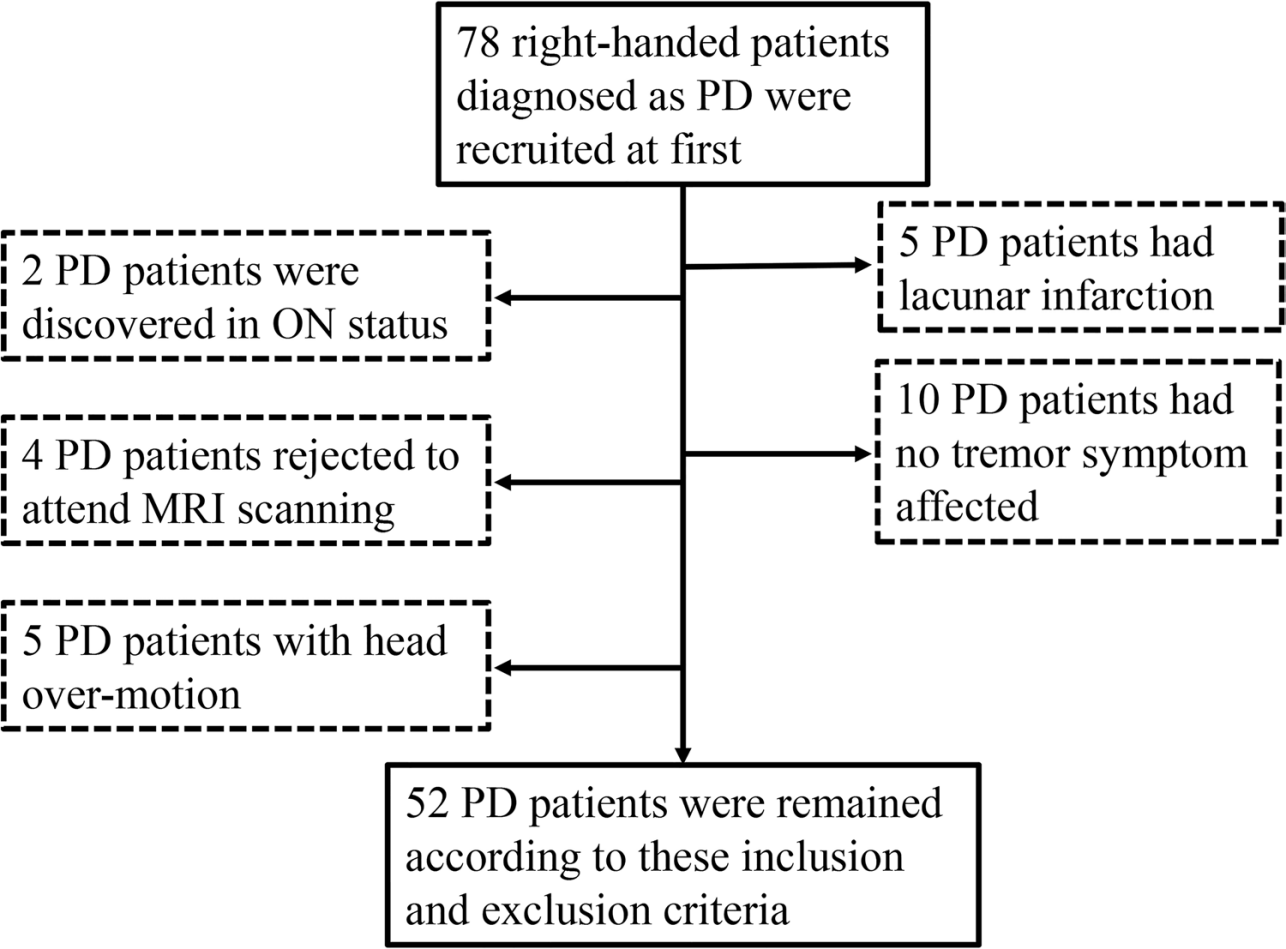
Flowchart of subject exclusion

Seventy-eight right-handed PD patients were prospectively recruited in this study. These patients were diagnosed by an experienced neurologist (B.Z.) according to the UK Parkinson’s Disease Society Brain Bank criteria (Hughes et al., 1992) and signed the informed consent forms in accordance with the approval of the Medical Ethics Committee of Second Affiliated Hospital of Zhejiang University School of Medicine. Subjects with a history of other psychiatric, neurologic or vascular disorders, brain trauma, or general exclusion criteria for MR scanning were excluded. Accordingly, five subjects were excluded due to the lacunar infarction (Fig. 1). Furthermore, for PD patients taking anti-parkinsonian drugs, all examinations were carried out after withdrawing all anti-parkinsonian medicine overnight (at least 12 h) to make sure they were in OFF status. Two subjects in ON status were excluded.

Clinical evaluations including age, gender, education, disease duration (from the day that parkinsonian symptoms occur to clinical evaluate), the total daily levodopa equivalent dose (LED) and the Unified Parkinson's disease rating scale motor part (UPDRS III) were recorded for all subjects. Specifically, for each patient, the motor symptoms were re-evaluated in ON-medication status defined as 1 h following anti-parkinsonian treatment (one tablet of immediate release carbidopa/levodopa 50/200 mg).

The score of each sub-symptom was calculated both in OFF and ON status, including tremor score: UPDRS III 20 + 21; rigidity score: UPDRS III 22; and bradykinesia score: UPDRS III 23 + 24 + 25 + 26 + 27 + 31. Ten subjects without tremor symptom affected were excluded. The DA responsiveness of tremor (simplified as tremor responsiveness) for each subject was calculated as follows:

$$\text{Tremor responsiveness (tremor change rate)=}{(OFF \;tremor\;score-ON\;tremor\;score)}/OFF\;tremor\;score$$

### MRI data acquisition and preprocessing

All subjects were scanned on a 3.0 T MRI scanner (GE Discovery 750) equipped with an 8-channel head coil. During MRI scanning, the heads of subjects were stabilized with a foam pad, the ears were plugged with earplugs to reduce the noise, and all subjects were told to keep stable with their eyes closed.

Structural T1-weighted images were acquired using a fast spoiled gradient recalled sequence: repetition time (TR) = 7.336 ms; echo time (TE) = 3.036 ms; inversion time = 450 ms; flip angle (FA) = 11°; field of view (FOV) = 260 × 260 mm^2^; matrix = 256 × 256; slice thickness = 1.2 mm; 196 continuous sagittal slices. Rs-fMRI images were acquired using a gradient recalled echo-echo planar imaging sequence: TR = 2000 ms; TE = 30 ms; FA = 77°; FOV = 240 × 240 mm^2^; matrix = 64 × 64; slice thickness = 4 mm; slice gap = 0 mm; 38 interleaved axial slices. After completing an initial rs-fMRI scanning in the OFF-medication status, PD patients were given a standard carbidopa/levodopa dose and re-scanned 1 h afterward in ON-medication status.

Specifically, four subjects who rejected to attend MRI scanning, and five subjects with higher head motion were excluded (> 2 mm in displacement or 2 degrees in rotation; or over 1/3 frames were defined as bad points (Power et al., 2012)). Subsequently, 52 subjects were obtained (Fig. 1) and were further analyzed.

The rs-fMRI data preprocessing was first performed using fMRIPrep v20.1.1 (https://fmriprep.org/en/stable/) (Esteban et al., 2019) as following: 1) each T1-weighted image was corrected for intensity non-uniformity and skull-stripped; 2) the brain surfaces were reconstructed using recon-all from FreeSurfer software; 3) the brain-extracted T1-weighted images were normalized to the ICBM 152 Nonlinear Asymmetrical template version 2009c 2 mm isotropic space through nonlinear registration; then, 4) the normalized T1-weighted images were segmented to cerebrospinal fluid, white matter and gray matter; 5) the functional data was corrected for slice-timing, motion distortion and susceptibility distortion, and afterwards registered to its own corresponding T1-weighted images using boundary-based registration with default 9 degrees of freedom. To be detailed, the susceptibility distortion correction (SDC) was performed with the fieldmap-less function imbedded in fMRIPrep. At first, a susceptibility distortion warp (corresponding displacement field) was estimated via nonlinear registration by using symmetric normalization (SyN) implemented in Advanced Normalization Tools (ANTs) and the average fieldmap atlas (https://fmriprep-test.readthedocs.io/en/latest/sdc.html#treiber2016). Then the warp was used to correct for susceptibility distortions (Esteban et al., 2019).

All processed rs-fMRI data was further manufactured by fMRIDenoise (https://github.com/compneuro-ncu/fmridenoise) with the default procedures, including temporal band-pass filtering (0.008–0.08 Hz), detrending, and nuisance covariates regression (including 24 head parameters, white matter and cerebrospinal fluid confounds, framewise displacement and DVARS regressors). After which, all functional data was smoothed with a 5 mm FWHM Gaussian kernel.

Afterwards, all preprocessed images were carefully examined for the regions that were sensitive to dephasing artifacts. And we found that all functional images had a decent quality and were qualified for further analysis.

### Functional connectivity processing

FC between dopaminergic subcortical nuclei and cerebral cortex as well as cholinergic subcortical nuclei and cerebral cortex were constructed both in OFF and ON status separately. At first, eight subcortical nuclei, including bilateral thalamus, caudate, putamen and pallidum from Harvard–Oxford subcortical atlas were acquired and defined as dopaminergic-related region of interests (ROIs). The cholinergic nuclei were defined according to the stereotaxic probabilistic maps which obtained from 10 postmortem brain using histological sections (Zaborszky et al., 2008). These maps were imbedded in the Statistical Parametric Mapping (SPM, https://www.fil.ion.ucl.ac.uk/spm/) toolbox Anatomy v22c. In case the cell clusters from different brains overlapped in one voxel, we used the maximum probability maps of BF, including bilateral BF 123 and bilateral BF 4. Then, the cerebral cortex was parcellated into 96 segments according to the Harvard–Oxford cortical atlas.

Afterwards, the time course of each ROI was extracted and the connectivity between subcortical ROI and cortical ROI was constructed by using Pearson correlation. Fisher’s r-to-z transformation was performed to improve the data’s normality. Accordingly, a dopaminergic network (96 × 8) and a cholinergic network (96 × 4) were obtained respectively. The FC between two ROIs was defined as an edge, and the connectivity strength of each edge was recorded. Then, the functional reactivity of each edge during levodopa administration was calculated as follows: (the spacing displayed for the formula was unmatched with the text)

$$Functional\;reactivity \left(FC\;change\;rate\right)=\frac{\text{ON connectivity strength-OFF connectivity strength}}{\text{abs (OFF connectivity strength)}},$$

where abs returns the absolute number. According to the formula, functional reactivity with positive value indicated that the connectivity strength was increased in ON status after levodopa administration, while negative value suggested that the connectivity strength was decreased in ON status.

### Imaging feature selection

**Fig. 2:**
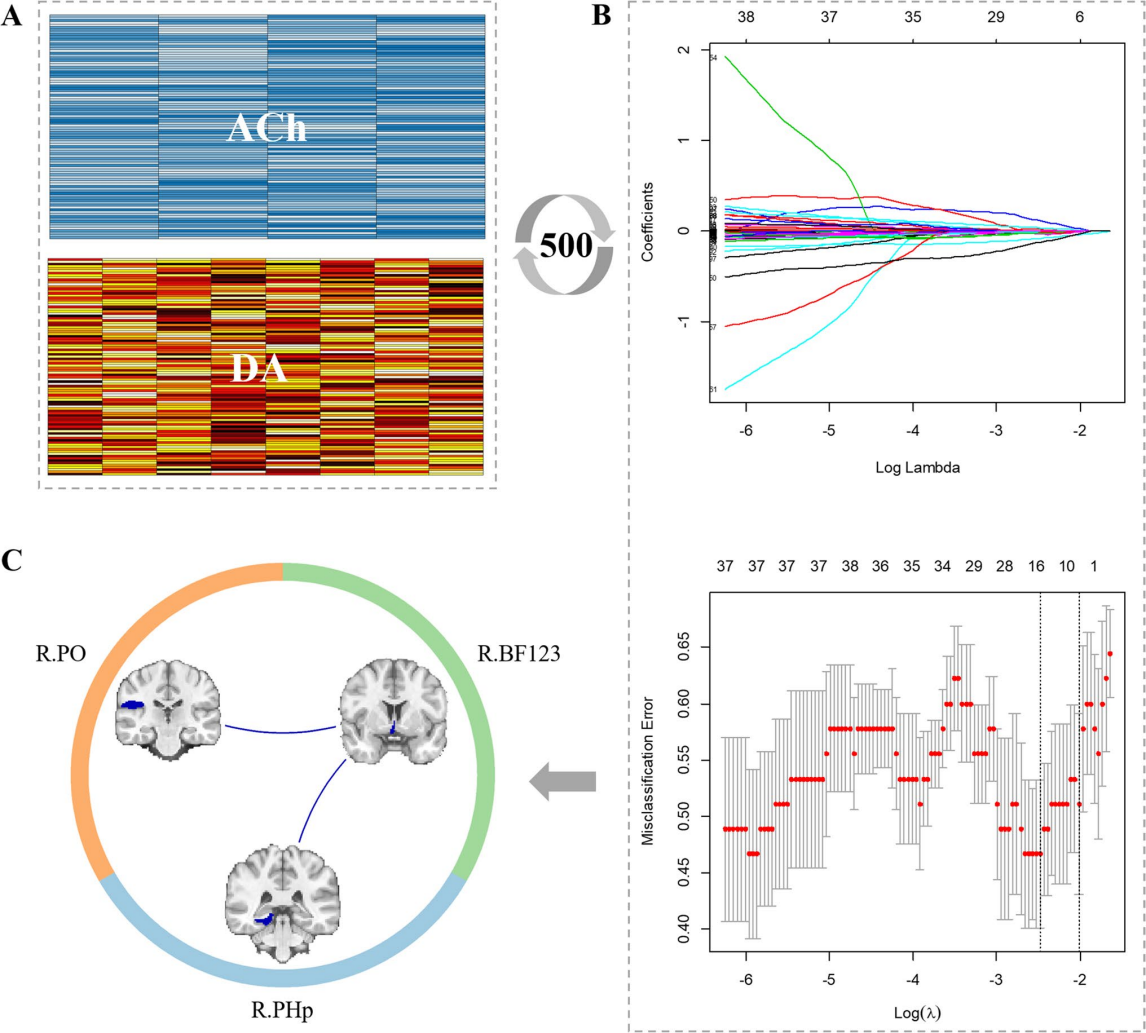
The imaging feature selection. A, the cholinergic connectivity matrix (96 × 4, ACh) and dopaminergic connectivity matrix (96 × 8, DA). B, the LASSO regression model construction, which was performed 500 times for ACh and DA separately. C, the final selected edges, including R.BF123-R.PHp and R.BF123-R.PO

First of all, the tremor responsiveness median score of total 52 subjects was obtained. Then, PD patients were divided into two groups according to the median score: DA-responsive tremor group with a tremor improvement above or equal to the median score, and DA-resistant tremor group with a tremor improvement below the median score. Accordingly, 24 DA-resistant tremor and 28 DA-responsive tremor patients were obtained. To identify the candidate edges with different functional reactivity during levodopa administration between PD groups, the functional reactivity of each edge was used as independent variables in least absolute shrinkage and selection operator (LASSO) regressions, and disease status (i.e., DA-resistant tremor or DA-responsive tremor) was regarded as the dependent variable. To be detailed, LASSO logistic regression was regularly used for variable selection to determine those that were particularly relevant for explaining the dependent variable (Juttukonda et al., 2019) and was performed by glmnet package (Friedman et al., 2010) imbedded in R Statistical Software (https://www.r-project.org/). The feature selection was performed for dopaminergic variables (96 × 8, 768 variables in total) and cholinergic variables (96 × 4, 384 variables in total) respectively (Fig. 2A). And each was performed on the sampled subjects (80% stratified sampling probability, without replacement) 500 times. The frequency of each edge being chosen was recorded. In detail, each selection involved a model construction and an inner tenfold cross-validation procedure (Fig. 2B). Then, the edges were considered probable for distinguishing two PD groups in ≥ 60% selection times. This cut-off selected the edges with probable likelihood of distinguishing two PD groups for further analysis.

To eliminate the potential tendentiousness induced by the separation of dopaminergic and cholinergic variables, feature selection was re-performed in all 1152 variables combined by dopaminergic and cholinergic variables. The processing procedure was the same as above.

### Statistical analyses

Demographic and clinical variables between groups were analyzed in Statistical Product and Service Solutions (SPSS version 25.0). The normal distribution of data was tested by the Kolmogorov–Smirnov test. Differences between groups were analyzed with two-sample t-test, Pearson chi-square, or Nonparametric tests appropriately. *P* < 0.05 was regarded as statistically significant.

To test the difference between groups for the selected edges from LASSO, group comparisons of functional reactivity were conducted by General Linear Model (GLM) with age, gender and education regressed out. Bonferroni correction was performed for the multi-comparisons (*P* < 0.05/2 = 0.025, as two edges were selected by LASSO in our study).

The edges with significantly different functional reactivity between PD groups were further analyzed as following. First, the group comparisons for their FC were explored by using GLM analysis both in OFF and ON status, with age, gender and education as covariates. The OFF–ON comparisons of FC were conducted by paired t-test or Wilcoxon Signed Rank Test with *p* < 0.05 as statistically significant.

Furthermore, Linear mixed-effect model was performed to explore the interaction effect between group (i.e., DA-resistant tremor or DA-responsive tremor) and medication status (i.e., OFF or ON) on functional reactivity. Specifically, the functional reactivity was corrected by age, gender and education first and then put to model construction. Partial correlation analysis was applied to explore the relationship between functional reactivity and tremor responsiveness with age, gender and education regressed out. *P* < 0.05 was regarded as significant.

### Complementary analyses

1) To clearly illustrate the alterations of significant edges under PD pathology, we explored the FC difference of these edges between PD subgroup (DA-resistant tremor group and DA-responsive tremor group) and age, gender and education-matched normal controls (NC) either in OFF status or ON status. These analyses were conducted by the GLM with age, gender and education regressed out. *P* < 0.05/2 = 0.025 was considered as significant.

2) Group comparisons of the overall dopaminergic and cholinergic network between PD and NC were analyzed to illustrate the general neurotransmitter alteration conditions: first, the averaged connectivity strength of dopaminergic network and cholinergic network (calculated as the mean value of DA-FC matrix and ACh-FC matrix) were compared between groups; *p* < 0.05/2 = 0.025 was thought to be significant. Then, the relative activity of these two kinds of networks, designated as the difference between mean ACh-FC and mean DA-FC, was compared between groups; *p *< 0.05 was regarded as statistically significant.

## Results

### Demographic and clinical variables

Demographic and clinical variables were shown in Table 1. No significant difference was found in age (*p* = 0.937), gender (*p* = 0.477), education (*p* = 0.296), disease duration (*p* = 0.920), or LED (*p* = 0.985) between DA-resistant tremor group and DA-responsive tremor group.

**Table 1:** The demographic and clinical variables

Clinical variables	DA-resistant tremor	DA-responsive tremor	p values
Num	24	28	-
Age (yrs.)	60.08 ± 9.63	59.88 ± 8.30	0.937^a^
Gender (M/F)	14/10	19/9	0.477^b^
Education (yrs.)	9.52 ± 4.31	8.21 ± 4.56	0.296^a^
Disease duration (yrs.)	5.15 ± 4.49	4.39 ± 2.75	0.920^c^
LED (mg)	459.43 ± 355.36	494.38 ± 415.10	0.985^c^
UPDRS III OFF	22.33 ± 16.78	23.46 ± 13.08	0.308^c^
UPDRS III ON	16.08 ± 13.24	12.75 ± 10.49	0.413^c^
p value of ON/OFF	** < 0.001**^d*^	** < 0.001**^d*^	-
Tremor OFF	4.58 ± 3.94	3.93 ± 3.27	0.480^c^
Tremor ON	2.92 ± 2.52	0.68 ± 1.09	** < 0.001**^c*^
p value of ON/OFF	**0.001**^d*^	** < 0.001**^d*^	**-**
Rigidity OFF	4.46 ± 4.01	6.36 ± 4.65	0.063^c^
Rigidity ON	2.88 ± 2.98	3.57 ± 4.26	0.787^c^
p value of ON/OFF	**0.001**^d*^	** < 0.001**^d*^	-
Bradykinesia OFF	8.92 ± 7.81	9.00 ± 5.99	0.600^c^
Bradykinesia ON	6.50 ± 6.41	5.21 ± 4.87	0.672^c^
p value of ON/OFF	** < 0.001**^d*^	** < 0.001**^d*^	-
Tremor change rate	0.27 ± 0.24	0.92 ± 0.13	** < 0.001**^c*^

^a^two sample t-test; ^b^Pearson Chi-square test; ^c^Mann-Whitney U test; ^d^Wilcoxon Signed Rank Test. * indicate the significant results

No significant difference of UPDRS III, rigidity, bradykinesia, or tremor score was observed between two PD groups in OFF status. In ON status, UPDRS III, rigidity, and bradykinesia score still showed no significant difference between groups, while the tremor score of DA-responsive tremor patients was much lower than DA-resistant tremor (*p* < 0.001). Moreover, the tremor responsiveness was higher in DA-responsive tremor compared with DA-resistant tremor patients (*p* < 0.001).

The levodopa administration markedly alleviated the overall symptoms for both DA-resistant tremor and DA-responsive tremor patients (Table 1), confirming that the DA-resistant tremor was not a consequence of general failure of levodopa effectiveness, e.g., gastrointestinal malabsorption.

### LASSO feature selection

Two cholinergic edges were selected by the LASSO regression with a frequency ≥ 60% for distinguishing DA-resistant tremor and DA-responsive tremor patients: connectivity of right basal forebrain 123 to right posterior division of para-hippocampal gyrus (R.BF123-R.PHp) (60.4%) and connectivity of right basal forebrain 123 to right parietal operculum cortex (R.BF123-R.PO) (83.4%) (Fig. 2C).

The R.BF123-R.PO (60.8%) was still identified when combining dopaminergic and cholinergic variables, which validated that the separation of dopaminergic and cholinergic variables would not induce potential tendentiousness in our study.

### Group comparisons

The functional reactivity of R.BF123-R.PO was quite different for two PD groups. The value of functional reactivity was negative for DA-responsive tremor patients while which was positive for DA-resistant tremor patients (*p* = 0.004). This difference suggested that the changing of R.BF123-R.PO was in the opposite direction for two PD groups during levodopa administration. The functional reactivity of R.BF123-R.PHp did not show any significant difference between PD groups (*p* = 0.042, uncorrected) (Table 2).

**Table 2:** The group comparisons of imaging variables

Imaging variables	DA-resistant tremor	DA-responsive tremor	p values
Part1 Group comparisons of FC change rate			
R.BF123-R.PHp change rate	2.34 ± 6.53	-1.15 ± 5.45	0.042^a^
R.BF123-R.PO change rate	7.45 ± 14.53	-1.32 ± 3.30	**0.004**^a*^
Part2 Group comparisons of R.BF123-R.PO			
R.BF123-R.PO OFF	0.11 ± 0.20	0.16 ± 0.17	0.523^a^
R.BF123-R.PO ON	0.17 ± 0.28	0.01 ± 0.24	**0.040**^a*^
p value of ON/OFF	0.475^c^	**0.003**^b*^	-

^a^GLM with age, gender and education regressed out; ^b^paired t-test; ^c^Wilcoxon Signed Rank Test. * indicate the significant results. Bonferroni correction was performed for Part1 (*p* < 0.05/2 = 0.025) and *p* < 0.05 was regarded as statistically significant for Part2

**Fig. 3:**
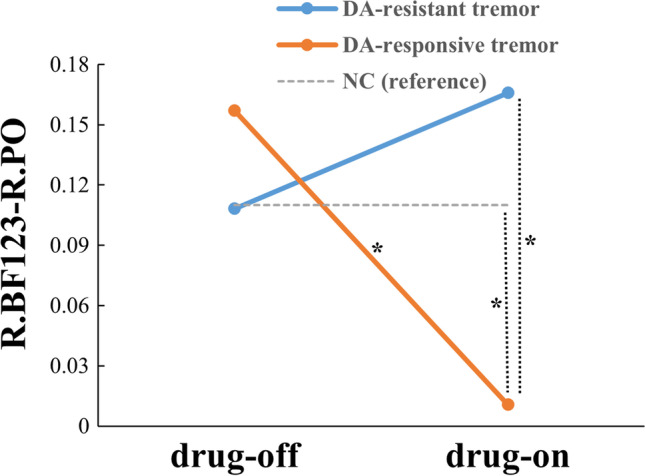
The FC alterations of R.BF123-R.PO in DA-resistant tremor and DA-responsive tremor patients regarding NC as reference. * indicate the significant results

Then, the FC alteration of R.BF123-R.PO was further analyzed. As a result, no statistical difference between two PD groups was observed in OFF status (*p* = 0.523); however, in ON status, a decreased FC was observed for DA-responsive tremor compared with DA-resistant tremor group (*p* = 0.040). Compared with OFF status, the FC in ON status was unchanged for DA-resistant tremor patients (*p* = 0.475), while for DA-responsive tremor subjects was decreased (*p* = 0.003) (Table 2, Fig. 3).

Specifically, an interaction effect of group × medication status was found for the functional reactivity of R.BF123-R.PO (*p* = 0.019).

### Correlation

**Fig. 4:**
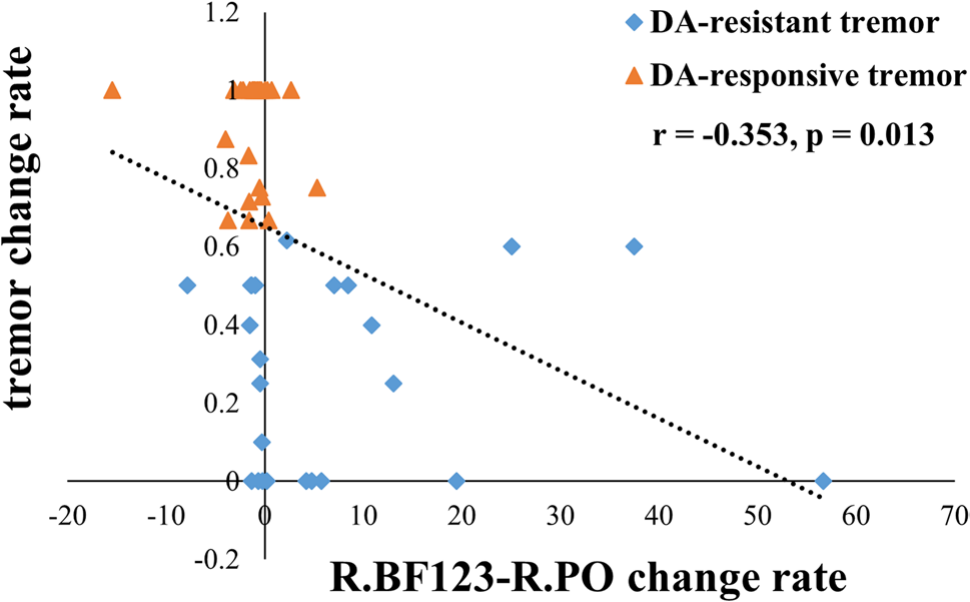
The correlation between R.BF123-R.PO change rate and tremor change rate

The FC change rate of R.BF123-R.PO was found to be negatively correlated with tremor change rate (r = -0.353, *p* = 0.013), which means that subjects with positive functional reactivity of R.BF123-R.PO during levodopa administration have poorer DA responsiveness of tremor, while patients with negative functional reactivity indicates a better DA responsiveness of tremor (Fig. 4).

#### Complementary analyses

1) The FC alteration of R.BF123-R.PO was further analyzed for two PD groups regarding NC as reference. As a result, no significant difference in OFF status was observed. In ON status, comparing with NC, the FC of R.BF123-R.PO was decreased for DA-responsive tremor patients (*p* = 0.038, uncorrected), while which was unchanged for DA-resistant tremor patients (*p* = 0.425) (Fig. 3, Table 3).

**Table 3:** The group comparisons of R.BF123-R.PO in PD subgroups regarding NC as reference

Variables	DA-resistant tremor	DA-responsive tremor	NC	p values
Num	24	28	93	-
Age (yrs.)	60.08 ± 9.63	59.88 ± 8.30	59.71 ± 6.34	0.999^a^
Gender (M/F)	14/10	19/9	42/51	0.084^b^
Education (yrs.)	9.52 ± 4.31	8.21 ± 4.56	9.74 ± 3.95	0.259^a^
R.BF123-R.PO OFF	0.11 ± 0.20	0.16 ± 0.17	0.11 ± 0.24	0.776^ci/^0.665^cii^
R.BF123-R.PO ON	0.17 ± 0.28	0.01 ± 0.24	0.11 ± 0.24	0.425^ci^/**0.038**^cii*^

^a^Kruskal-Wallis test; ^b^Pearson Chi-square test; ^c^GLM with age, gender and education regressed out; i, test between DA-resistant tremor and NC; ii, test between DA-responsive tremor and NC. * indicate the significant results with *p* < 0.05, ** indicate the significant results after Bonferroni correction with *p* < 0.05/2 = 0.025 (no significant result remained)

2) For the comparisons of overall dopaminergic and cholinergic network between PD and NC, we found that the averaged connectivity strength of dopaminergic network was decreased in PD compared with NC in OFF status (*p* < 0.001). While no significant difference was found after levodopa administration (*p* = 0.081) (Table 4). These results indicated that the dopaminergic activity was decreased under PD pathology and was restored after DA supplementing. However, no significant difference between PD and NC was found for the averaged connectivity strength of cholinergic network either in OFF status (*p* = 0.099) or ON status (*p* = 0.074).

**Table 4:** The group comparisons of overall dopaminergic and cholinergic network, and the difference between these two kinds of networks

Variables	PD	NC	p values
Part1 Group comparisons of mean DA-FC and ACh-FC			
DA-FC OFF	0.25 ± 0.13	0.32 ± 0.14	** < 0.001**^a*^
DA-FC ON	0.29 ± 0.16	0.32 ± 0.14	0.081^a^
p value of ON/OFF	0.189^c^	-	-
ACh-FC OFF	0.18 ± 0.11	0.21 ± 0.14	0.099^a^
ACh-FC ON	0.18 ± 0.11	0.21 ± 0.14	0.074^a^
p value of ON/OFF	0.450^b^	-	-
Part2 Group comparisons of the difference between ACh-FC and DA-FC			
difference value OFF	-0.07 ± 0.12	-0.11 ± 0.12	**0.032**^a*^
difference value ON	-0.11 ± 0.13	-0.11 ± 0.12	0.879^a^
p value of ON/OFF	0.075^b^	-	-

^a^GLM with age, gender and education regressed out; ^b^Wilcoxon Signed Rank Test; ^c^paired t-test; difference value was designated as mean ACh-FC subtracts mean DA-FC; * indicate the significant results. Bonferroni correction was performed for Part1 (*p* < 0.05/2 = 0.025) and *p* < 0.05 was regarded as statistically significant for Part2

Furthermore, we found that the difference value between ACh-FC and DA-FC was increased in PD comparing with NC in OFF status (*p* = 0.032). While no significant difference was found in ON status (*p* = 0.879) (Table 4). This finding verified that the imbalanced activity between cholinergic and dopaminergic network existed in PD, exhibiting as ACh overactive relative to DA, and could be restored after DA administration.

## Discussions

The differed tremor responsiveness is an important feature for PD. In this study, we made a comprehensive exploration on the alterations of dopaminergic and cholinergic connectivity during levodopa administration to uncover the mechanisms of differed tremor responsiveness in PD. The main findings were as follows: 1) the reactivity of cholinergic FC to levodopa showed the highest possibility to distinguish DA-resistant tremor and DA-responsive tremor patients; 2) the FC alteration of R.BF123-R.PO during levodopa administration between two PD groups was quite different, which remained unchanged for DA-resistant tremor patients but was decreased for DA-responsive tremor after levodopa administration; and 3) the functional reactivity of R.BF123-R.PO was further found to be negatively correlated with the tremor responsiveness.

### Cholinergic system is important for the distinct tremor responsiveness in PD

The reactivity of two cholinergic edges to levodopa were selected with the high probability distinguishing DA-resistant tremor and DA-responsive tremor patients. Although levodopa replacement therapy could restore the dopaminergic deficiency, the reactivity of dopaminergic connectivity could not distinguish two PD groups while cholinergic connectivity could. These findings indicated that the levodopa did exert an influence on the inner cholinergic system as previous studies suggested (Geula & Slevin, 1989; Napier & Potter, 1989), and more deeply, the cholinergic system played an important role in differed tremor responsiveness in PD.

It is reported that the severity of parkinsonian tremor (including resting tremor and action tremor), unlike bradykinesia, rigidity or postural abnormalities, was not related to the degree of dopaminergic denervation (Benito-Leon et al., 2018). Furthermore, the anticholinergics were found to be preferentially efficient for tremor compared with other parkinsonian symptoms (Cantello et al., 1986; Koller, 1986). The above evidence suggested that the cholinergic system is important for the parkinsonian tremor (Connolly & Lang, 2014; Lim et al., 2009). The close relationship of cholinergic system with tremor, combining its distinguishable characteristic suggested by our study indicated that the different cholinergic reactivity to levodopa may be an underlying mechanism for the differed tremor responsiveness in PD.

### Cholinergic reactivity of parietal operculum is distinct among PD patients with differed DA responsiveness of tremor

Alterations of the connectivity of R.BF123-R.PO exhibited a quite different pattern between two PD groups: the connectivity remained unchanged for DA-resistant tremor patients after levodopa administration while for DA-responsive tremor patients was decreased. This finding suggested that the reduced cholinergic activity may be an important characteristic for the responsive tremor in PD, and further, the distinct cholinergic reactivity of parietal operculum would be a core pathophysiology for the distinct DA responsiveness of parkinsonian tremor.

The overactivated cholinergic activity was reported to be related to the motor deficit in PD (Cools et al., 1975; McKinley et al., 2019; Ztaou & Amalric, 2019). In this study, we found that the relative activity between cholinergic and dopaminergic network was increased under PD pathology. It is reported that the relative overactivity of cholinergic function is because dopamine deficiency accompanies a smaller reduction in ACh availability (McKinley et al., 2019), or the degeneration of dopaminergic nigrostriatal neurons elicits cholinergic neurons to sprout (Spehlmann & Stahl, 1976). Overall, the relatively overactive cholinergic connectivity is an important pathological marker for PD.

The parietal area is a core region where the anticholinergic medication functioned on (P. H. Lee et al., 2008), which suggests that the parietal operculum is under cholinergic control and is closely related to cholinergic modulation. The parietal operculum contains secondary somatosensory area and is densely connected to both somatosensory and motor areas, linking it to sensorimotor integration and motor control (Eickhoff et al., 2010; Jackson et al., 2011), which plays an important role in the motor performance for PD (Nishida et al., 2021; Sharman et al., 2013). Previous studies indicated that the overactivated parietal operculum was related to severer motor dysfunctions in PD (Herz et al., 2014; Nishida et al., 2021). In detail, a meta-analysis of 24 functional neuroimaging studies demonstrated that the parietal area is relatively overactivated during motor execution or imagery in PD patients (Herz et al., 2014), and another study revealed that the reduced activity of parietal operculum could alleviate the motor disfunctions in PD (Nishida et al., 2021). All of these indicated that after levodopa administration, the reduced activity of parietal operculum which was under cholinergic innervation was an important characteristic for the DA-responsive tremor in PD.

The cholinergic reactivity of parietal operculum was further found to be negatively correlated with the tremor responsiveness, which suggested that the parietal operculum is an important regulatory for parkinsonian tremor. Previous studies reported that the parietal operculum was correlated with tremor amplitude-related activity in PD and had distinct activation characteristics among PD patients with differed tremor responsiveness (Dirkx et al., 2019). Moreover, the thickness of inferior parietal gyrus which overlapped with parietal operculum in a portion was found to be associated with tremor severity in PD (Benito-Leon et al., 2018), and the secondary somatosensory cortex was discovered as an important node in the tremor related oscillatory network through magnetometer (Pollok et al., 2009). Secondly, this significant relationship strengthened that the alteration of cholinergic connectivity was quite different between two PD groups with distinct tremor responsiveness and indicated that the distinct cholinergic reactivity of parietal operculum during levodopa administration may be an underlying mechanism for the differed DA responsiveness of tremor in PD.

### Limitations

Several limitations of this study should be acknowledged. First, the sample size of this study was relatively small, further prospective studies with a larger sample size are needed to validate these findings. Second, the rs-fMRI could not directly exhibit the neurotransmitter-related alterations in brain but an indirect measure, even so, it is an important technique for exploring the neurotransmitter-related alterations in vivo. Additionally, PD patients in this study were under antiparkinsonian treatment and withdrew from levodopa for 12 h, which may have a residual long-duration effect of levodopa. Although the minimum 2-week duration was suggested as the washout period for PD patients eliminating the symptomatic effects from levodopa (Fahn et al., 2004; Hauser et al., 2000), the long washout period was hard to control for drug-managed PD patients. Thus, we just urged our PD patients withdrawing antiparkinsonian medicine overnight, which is related to a short-duration effect of dopamine (Cilia et al., 2020; Muenter & Tyce, 1971). Future studies with drug-naïve PD patients employed could be used to validate our results. Furthermore, the split of PD subjects according to their median score of tremor change rate may be less rigorous, because those who near the median score may be similar in their tremor response to dopamine. But as former studies exhibited (Kagerer et al., 2020; Nicolas et al., 2020) that using median score as boundary to analyze the effect of one factor was an alternative way.

## Conclusions

This study revealed the differential reactivity of cholinergic connectivity between basal forebrain and parietal operculum for PD patients with differed DA responsiveness of tremor: only patients with DA-responsive tremor exhibited decreased connectivity after levodopa administration. These findings underscored that the reduced cholinergic connectivity of parietal operculum may be an underlying mechanism for the responsive tremor in PD and the distinct cholinergic reactivity of parietal operculum to levodopa may be a core pathophysiology for the differed DA responsiveness of tremor in PD, which may sprout a new direction for the future therapeutic treatment for PD patients.

## Data Availability

The data and material used and/or analyzed during the current study are available from the corresponding author on reasonable request.

## Code availability

The software used in this study was available on websites and the customed code was available from the corresponding author on reasonable request.
